# Development of a multi-point mapping protocol for myotonometric assessment: a methodological pilot study

**DOI:** 10.1038/s41598-025-34869-5

**Published:** 2026-01-07

**Authors:** Luiz Henrique Cabral Duarte, Larissa Sinhorim, Julya Charara Aires da Silva, Giovanna Guolo Coutinho, Luis Mochizuki, Graziela Morgana Silva Tavares, Robert Schleip, Iramar Baptistella do Nascimento, Gilmar Moraes Santos

**Affiliations:** 1 Universidade do Estado de Santa Catarina, Florianópolis, Brazil; 2 Universidade de São Paulo, São Paulo, Brazil; 3 Universidade Federal do Pampa, Uruguaiana, Brazil; 4 Technical University of Munich, Munich, Brazil

**Keywords:** Soft tissue, Connective tissue, Muscle, Fascia, Myotonometry, MyotonPRO, Anthropometry, Stiffness, Anatomy, Biomarkers, Health care, Medical research

## Abstract

The assessment of biomechanical and viscoelastic properties of superficial tissues using myotonometry has gained prominence in clinical and research settings due to its ease of use and ability to provide objective measurements. However, there is a tendency to assess only a single point without considering anatomical or functional variations, and its representativeness for broad regions, such as the lower limbs, remains uncertain. Consequently, we aimed to develop a standardized multi-point protocol for evaluating biomechanical and viscoelastic properties in the lower limbs using myotonometry, test its feasibility and discriminative capacity, and present preliminary exploratory findings. A multi-point myotonometric assessment protocol was developed, where thirty-eight assessment points were identified and described for evaluating the anterior, lateral, posterior, and inferior compartments of the lower limbs. The protocol was applied to both limbs of 13 healthy adult males (age: 32.15 ± 6.50 years). Values were described using mean and standard deviation, and comparisons between consecutive points were tested using the Wilcoxon signed-rank test. Effect sizes were calculated as r = Z/√N. IBM^®^ SPSS Statistics 20.0 was used with a significant level of 5%. Stiffness values (N/m) showed statistically significant variations between consecutive points, ranging from 136.87 N/m (± 25.55) to 1118.28 N/m (± 357.84), with effect sizes ranging from *r* = 0.595 to *r* = 0.874. The statistically significant increase in the proximal-distal and medio-lateral directions, with progressively higher values ​​in the distal and lateral regions, indicates a directional behavior of this property. The proposed multi-point methodology represents an advancement in non-invasive biomechanical assessment. As a methodological development study with preliminary findings, the protocol demonstrates capacity to detect location-dependent variations in stiffness with large effect sizes. Following validation in larger, diverse cohorts, it offers potential for improving diagnosis, therapeutic monitoring, and understanding tissue adaptation mechanisms in clinical and functional contexts.

**Trial registration: **CEPSH/UDESC (CAAE 65601722.5.0000.0118).

**Supplementary Information:**

The online version contains supplementary material available at 10.1038/s41598-025-34869-5.

## Introduction

Myotonometry has been established as a non-invasive, valid, operational simplicity, and reproducible tool for the quantitative assessment of the biomechanical and viscoelastic properties of soft tissues in clinical and research settings^1–3^. This technique enables precise measurement of tissue oscillation frequency (Hz), facilitating the analysis of biomechanical properties such as stiffness (N/m) and decrement (arb), as well as viscoelastic properties, including relaxation time (ms) and creep (arb)^4,5^.

Recent studies have demonstrated its efficacy in assessing various anatomical structures across different populations and health conditions^1–3,6–9^, establishing it as a reliable instrument for quantifying tissue properties. However, methodological standardization remains a critical area of investigation, particularly because existing protocols primarily originate from three key sources: the methodology established by Bizzini and Mannion^4^, the operational guidelines of MyotonPRO (Myoton^®^ AS, Estonia) outlined by Gapeyeva and Vain^5^, and subsequent research that has adapted and refined these foundational references^6,10^.

There is a methodological tendency in literature to assess a single measurement point within the structure of interest, typically positioned at its center, due to reliance on primary references. However, variations in size, anatomical location, biological composition, and functional role of the evaluated structures may lead to location-dependent variations that might not be captured by single-point assessments^2–6,10–15^. Unlike standardized parameters such as the number of mechanical taps performed per measurement, for example^4,5,16^, it remains untested whether a single measurement point is sufficient to reliably characterize viscoelastic and biomechanical properties in broad and structurally diverse anatomical regions, such as those found in the lower limbs.

Seeking to advance this understanding, researchers have explored new methodological approaches. Recent studies have evaluated multiple measurement points within the same structure, demonstrating that tissue properties can vary significantly depending on the specific location of measurement^7,8,17–21^. However, the lack of standardized, reproducible protocols and the methodological complexity of multi-point evaluations have hindered their widespread adoption in clinical and scientific practice.

The present investigation represents a methodological development study with an exploratory cross-sectional component. The primary objective is to develop and validate the feasibility of a standardized, reproducible multi-point assessment protocol for myotonometry in lower limbs, addressing current gaps in measurement standardization. The secondary objective is to generate preliminary exploratory data on stiffness distribution patterns to inform future hypothesis-driven research. We emphasize that findings should be interpreted as hypothesis-generating rather than definitive, requiring systematic validation in larger, adequately powered studies before clinical application or establishment of normative reference values.

Therefore, the present study aims to develop a standardized and reproducible measurement protocol for assessing biomechanical and viscoelastic properties of the lower limb via myotonometry. Additionally, as a methodological feasibility study, we seek to generate preliminary exploratory data on myotonometry variables and investigate the behavior of stiffness (N/m)—the primary myotonometric variable in research and clinical settings—to demonstrate the protocol’s capacity to detect statistically significant variations across multiple measurement points.

## Methods

### Study design

This methodological study, incorporating data from a cross-sectional observational pilot study design, aims to develop a multi-point assessment protocol for evaluating viscoelastic and biomechanical properties of the lower limb via myotonometry. The protocol was designed with a primary focus on key muscular and tendinous structures, ensuring alignment with current biomechanical and clinical research to facilitate implementation, comparison, and replication across different studies.

The study was approved by the Human Research Ethics Committee of Santa Catarina State University (Brazil) on September 27, 2023 (Opinion No. 5.830.520, CAAE: 65601722.5.0000.0118) and included 13 male participants aged 25 to 44 years. The modest sample size was determined by feasibility constraints for this initial protocol development phase, including the complexity and participant burden of assessing 38 anatomical locations per visit, and the project’s exploratory, proof-of-concept nature, with the primary aim being demonstration of the protocol’s discriminative capacity rather than establishment of population-level normative values. All methods were performed in accordance with the relevant guidelines and regulations, all participants were informed of the collection procedures, agreed to participate in the study and signed an informed consent form.

### Participants

In order to test the applicability and gather initial data regarding the newly proposed approach, a cross-sectional observational pilot study was conducted. The inclusion criteria for this pilot study sample were: healthy adult men (25 to 44 years old), agreement to sign the informed consent form, willingness to collaborate, and availability to participate in data collection.

The exclusion criteria were: female participants, individuals outside the specified age range, those with severe cardiovascular disease, neuromuscular disorders, or any other health conditions preventing safe execution of the assessments, history of recent lower-limb surgery, reports of acute or chronic lower-limb pain, dependence on assistive devices for locomotion, or severe cognitive impairment. The inclusion and exclusion criteria were assessed using the subject characterization form. All participants included in the sample met the established criteria for this study. The decision to evaluate only men aged 25 to 44 years was made primarily because this was a pilot study with a smaller scope, but also because previous studies^6,17,20^ have indicated that myotonometry measurements may vary significantly between individuals of different sexes and age groups.

Post-hoc power analysis were performed in GPower 3.1.9.7 for the Wilcoxon signed-rank test (matched pairs, two-tailed, α = 0.05, *n* = 13) using observed effect sizes. To represent the typical magnitude of our findings, we anchored the analysis on the median effect size (r̃ = 0.834, SD = 0.09) and conducted a sensitivity analysis spanning the empirical range (*r* = 0.595–0.874). Results for three representative scenarios are summarized in Supplementary Table S1 and detailed in Supplementary Figure S1. These power estimates employ standard parametric approximations for non-parametric effect sizes, an approach validated in methodological literature [22–24]. While such conversions involve inherent approximations, the substantive conclusions remain robust: observed effects consistently exceed conventional thresholds for large effects across all measurement comparisons.

While achieved power at the median and upper-bound effects was substantially higher than at the lower bound, the limited sample size and intra-subject dependence warrant caution. The achieved power of 0.483 at the lower bound (*r* = 0.595) corresponds to the minimum effect size observed, which was atypical among our comparisons. The median effect size (r̃=0.834, achieved power = 0.765) more accurately represents the protocol’s typical discriminative capacity. All statistical tests yielded *p* < 0.005, with the majority achieving *p* < 0.001, indicating robust statistical evidence beyond any single power metric. Prospective, sample size calculations indicate that for large effects (*r* ≥ 0.80, similar to our observed median), *n* = 13 pairs achieve adequate power. However, we recommend ~ 26–30 independent participants to ensure robustness across potentially more heterogeneous populations and to enable subgroup analyses by demographic factors.

### Instrumentation

Data collection was conducted using three primary instruments: (1) an characterization assessment form (2) a MyotonPRO myotonometer (Myoton^®^ AS, Estonia) for Frequency (Hz), Stiffness (N/m), Decrement (arb), Relaxation (ms) and Creep (arb) evaluation, and (3) an InBody770 bioimpedance analyzer (InBody^®^ CO., South Korea) to assess body composition.

### Data collection procedure

The assessments were conducted by two previously trained evaluators. The first evaluator was responsible for administering the characterization questionnaire and collecting body composition data using the InBody770 bioimpedance analyzer (InBody^®^ Co., South Korea), while the second evaluator was responsible for applying the Multi-point Mapping Protocol and collecting myotonometry measurements at each proposed point using the MyotonPRO (Myoton^®^ AS, Estonia).

Measurements were performed following a standardized positioning protocol, with participants lying on an examination table in the supine position for marking and assessment of points on the anterior and lateral views, and in the prone position for marking and assessment of points on the posterior and inferior views. After marking the multi-point mapping protocol on both lower limbs, a 10-min rest period in the assessment posture (supine or prone) was established to minimize the influence of prior muscle activity.

During the resting and assessment periods, participants remained lying in the supine position for evaluation of the anterior and lateral points, and in the prone position for evaluation of the posterior points and plantar fascia. No pillow or comfort device was provided during the postures. However, to maintain slight knee flexion in both positions, a firm cushion (15 cm in diameter) was placed under the knees in the supine position and under the ankles in the prone position during marking and assessment. The examination table used had an opening that allowed participants to position their face comfortably, avoiding discomfort.

### Protocol development

The development of the protocol followed a consistent logical framework, beginning with a central measurement and incorporating additional measurement locations along the longitudinal axis of the structure of interest.

**Fig. 1:**
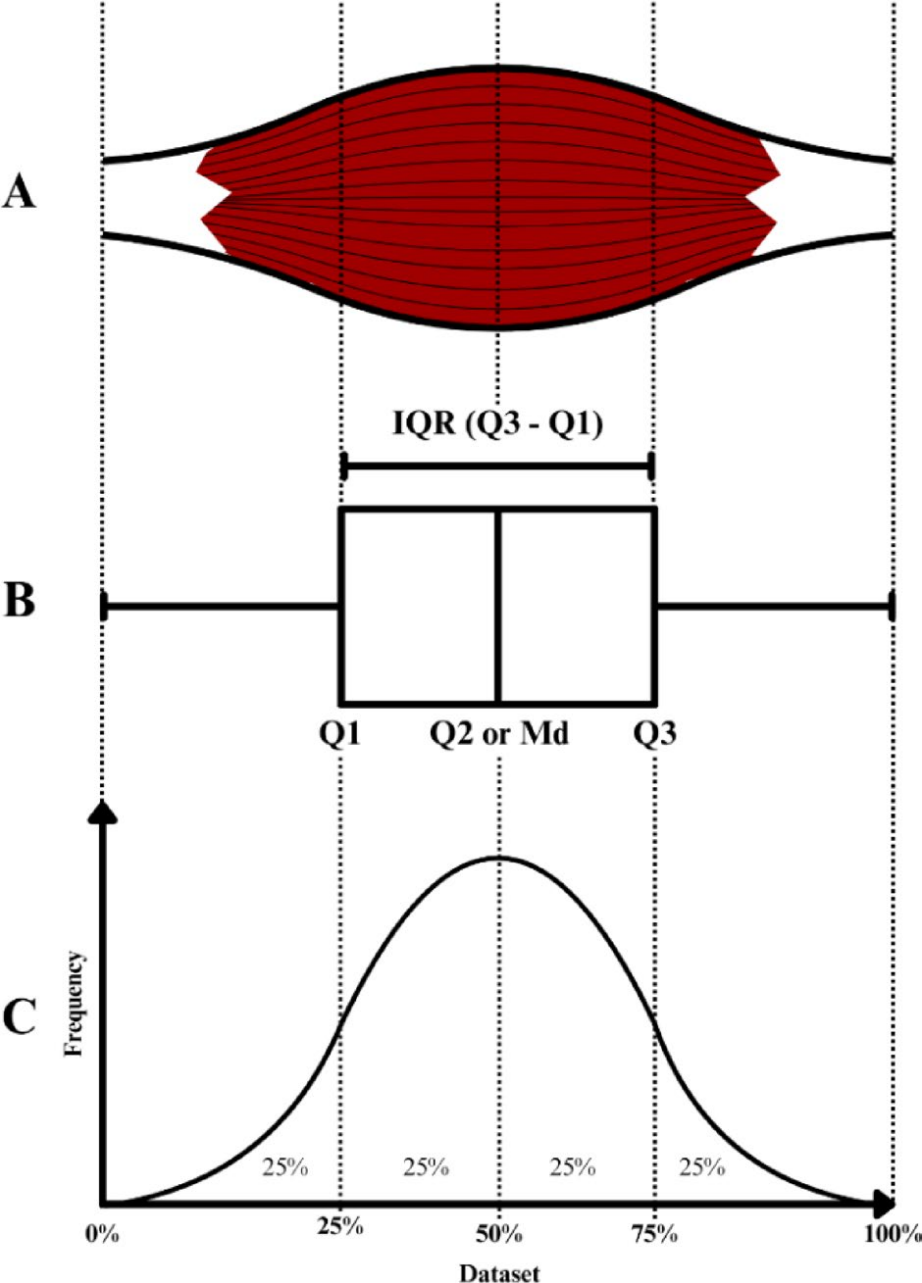
Representation of the points marked for evaluation in a generic muscle using the concept of median and interquartile range. (**A**) Generic muscle structure; (**B**) Boxplot of the interquartile range of the data set in Fig. 1C; (**C**) Fictitious data set with normal distribution ordered by frequency and markings referring to the points of interest of the interquartile range and median; IQR – Interquartile range; Q1 – First quartile; Q2 – Second quartile; Q3 – Third quartile.;

The protocol was grounded in statistical principles of central tendency and variability, utilizing the median and interquartile range as its theoretical basis^25^. It was initially established using the statistical concept of quartiles, which divides a structure into four equal segments, defining measurement points at 25%, 50%, and 75% of the total length, that was demonstrated in Fig. 1.

This concept was later expanded to enable a more detailed structural analysis, employing varied division ratios (1/2, 1/4, 1/6, and 1/8) based on specific anatomical and functional requirements. For example, larger and more structurally complex tissues, such as the biceps femoris, were divided into six equal segments (1/6) to provide a more detailed analysis of stiffness variations along its length. Conversely, smaller anatomical structures were assessed using simpler division schemes, such as quarters (1/4) or halves (1/2), ensuring that the measurement approach remained proportional to the anatomical and functional characteristics of each tissue.

**Fig. 2:**
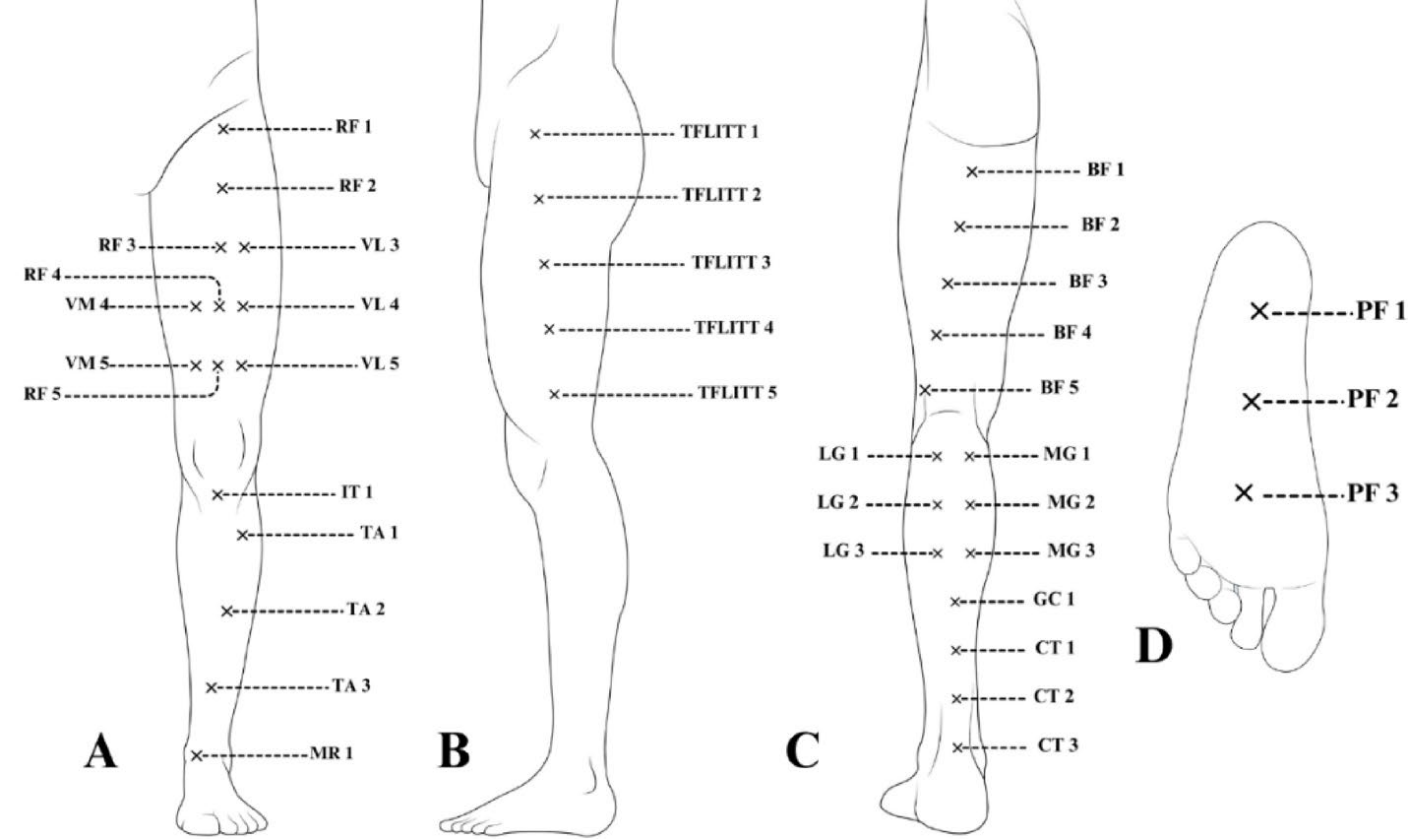
Summary and identification of the elaborated assessment points. (**A**) Anterior view; (**B**) Lateral view; (**C**) Posterior view; (**D**) Inferior view;

The protocol established 38 assessment points distributed among the main structures of the lower limbs: rectus femoris (RF) 5 points, vastus lateralis (VL) 3 points, vastus medialis (VM) 2 points, infrapatellar tendon (IT) 1 point, tibialis anterior (TA) 3 points, medial retinaculum (MR) 1 point, tensor fasciae latae and iliotibial tract (TFLITT) 5 points, biceps femoris (BF) 5 points, lateral gastrocnemius (LG) 3 points, medial gastrocnemius (MG) 3 points, musculotendinous junction between the gastrocnemius and calcaneal tendon (GC) 1 point, calcaneal tendon (CT) 3 points, and plantar fascia (PF) 3 points. Figure 2 summarizes the assessment points included in the protocol, while a detailed description of the locations and measurement procedures is provided in the results section.

### Assessment protocol for the anterior thigh region

**Fig. 3:**
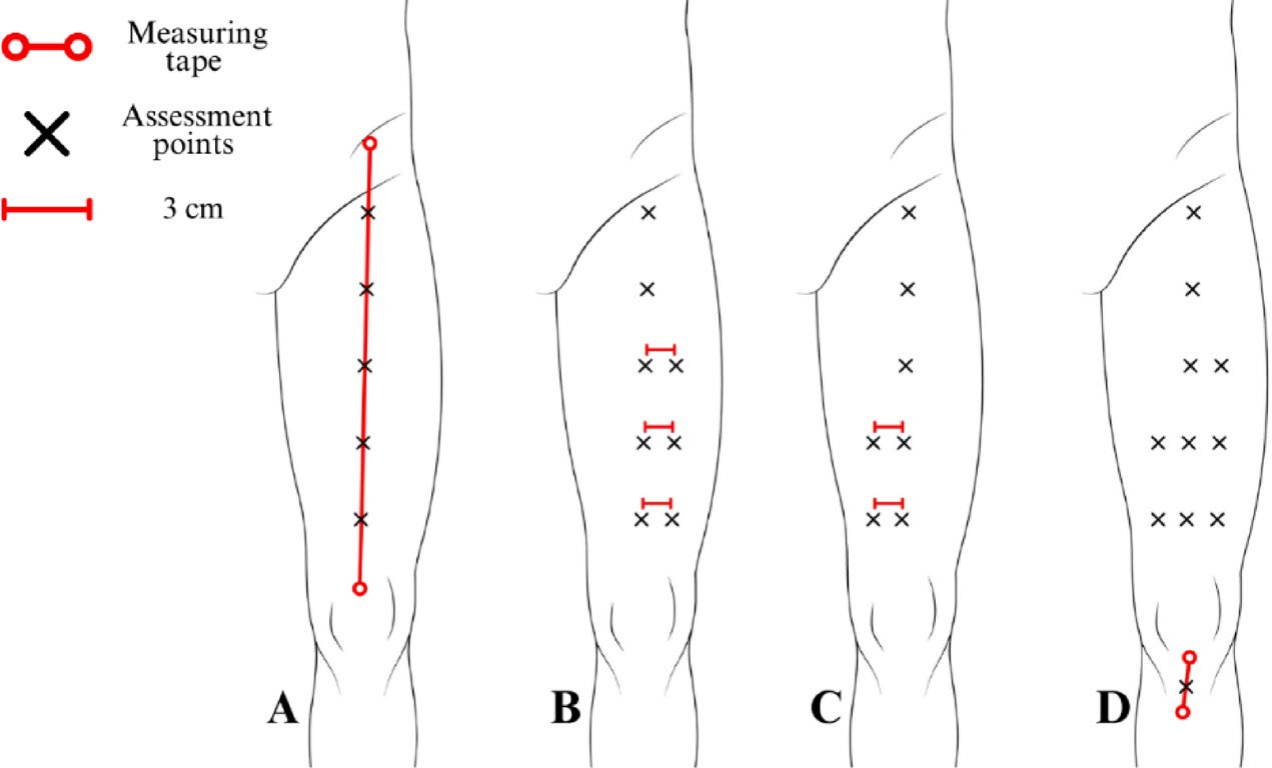
Protocol for mapping assessment points in the anterior thigh region. The figure presents the assessment protocol for the anterior thigh region, depicting the anterior view of the right lower limb. (**A**): Reference points are established at the anterior superior iliac spine (proximal reference) and the center of the base of the patella (distal reference), with rectus femoris assessment points positioned at five equidistant intervals along the total distance between these references. (**B**): Vastus lateralis assessment points are positioned three centimeters laterally from the three most distal rectus femoris points (RF3, RF4, and RF5). (**C**): Vastus medialis assessment points are positioned three centimeters medially from the two most distal rectus femoris points (RF4 and RF5). (**D**): The infrapatellar tendon assessment point is positioned at the midpoint between the inferior angle of the patella and the tibial tuberosity, representing the center of the tendon.

Using a measuring tape positioned between the anterior superior iliac spine and the center of the superior edge of the patella, the distance between these reference points was divided into six equal intervals, with markings placed at five equidistant points along this axis (Fig. 3A).

These five marked points made between the anterior superior iliac spine and the center of the superior edge of the patella correspond to the rectus femoris assessment locations, serving as additional reference points for the vastus lateralis and vastus medialis assessments. The vastus lateralis assessment points were derived from the three distal rectus femoris points (RF3, RF4, and RF5), with markings positioned three centimeters laterally (Fig. 3B).

Similarly, the vastus medialis assessment points were determined using the two most distal rectus femoris points (RF4 and RF5), where markings were placed three centimeters medially (Fig. 3C). Meanwhile, the infrapatellar tendon assessment point was positioned at the center of the tendon, precisely at 50% of the distance between the inferior angle of the patella and the tibial tuberosity (Fig. 3D).

### Assessment protocol for the anterior leg region

**Fig. 4:**
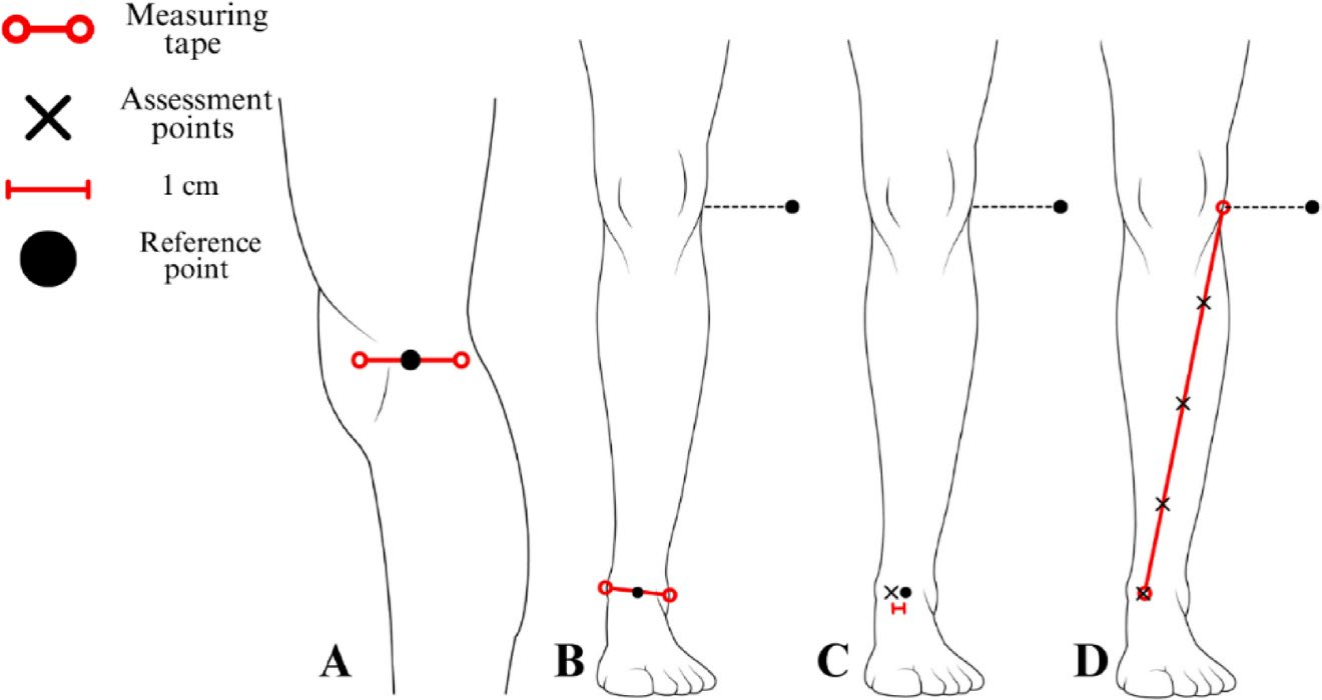
Protocol for mapping assessment points in the anterior leg region. The figure presents the assessment protocol for the anterior leg region, depicting the lateral and anterior view of the right lower limb. (**A**): The proximal reference point is established at the center of the lateral aspect of the knee joint line. (**B**): The reference point for the medial retinaculum (MR1) assessment is positioned at 50% of the distance between the malleoli. (**C**): The distal assessment point MR1 is located one centimeter medially from the center of the distance between the malleoli. (**D**): Reference points are established at the center of the lateral aspect of the knee joint line (proximal reference) and at the MR1 assessment point (distal reference), with tibialis anterior assessment points positioned at three equidistant locations, obtained by dividing the total distance into four equal intervals.

Initially, the first reference point was established at the center of the lateral aspect of the knee joint line (Fig. 4A). The second reference point, which also served as the assessment location for the medial retinaculum (MR1), was determined using the distance between the malleoli, where a reference point was marked (Fig. 4B). The actual medial retinaculum assessment point was then positioned one centimeter medially from the center of the malleoli (Fig. 4C).

A measuring tape was aligned between the knee joint line reference point and the medial retinaculum assessment point (MR1). The distance between these reference points was divided into four equal intervals, with three equidistant markings placed between them, corresponding to the tibialis anterior assessment points (Fig. 4D).

### Assessment protocol for the lateral thigh region

**Fig. 5:**
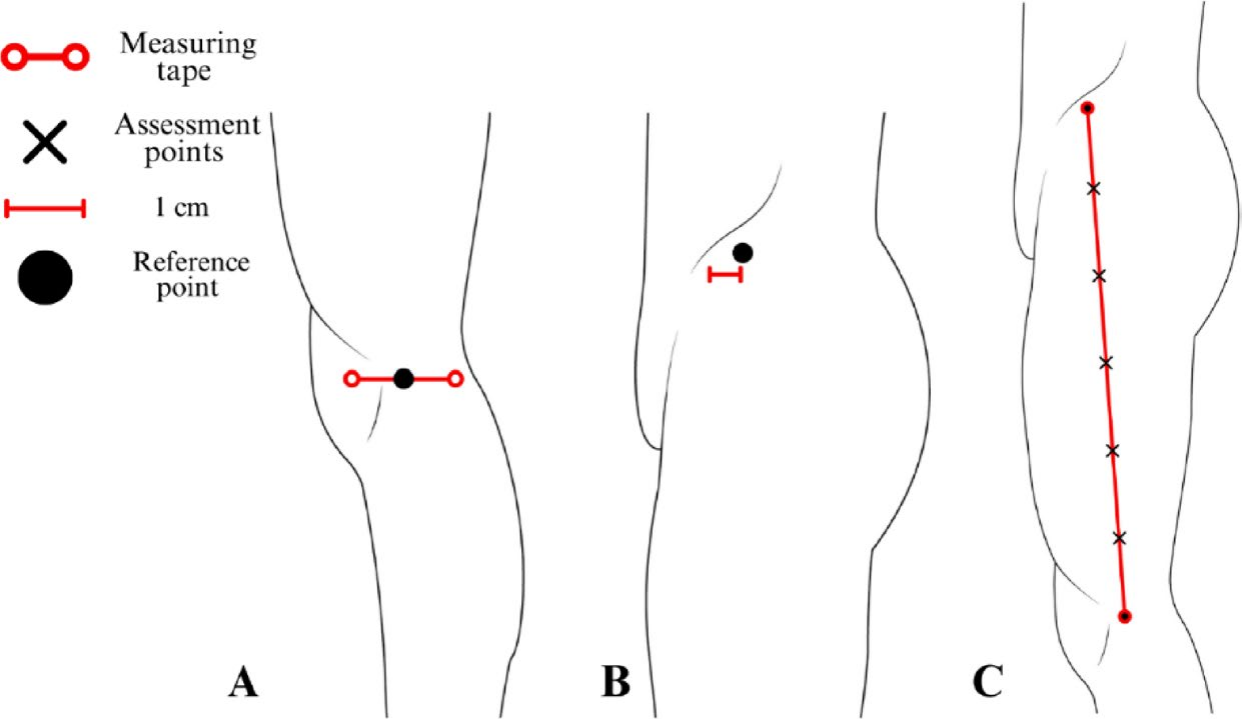
Illustration of reference and assessment points in the lateral thigh region. The figure presents the assessment protocol for the lateral thigh region, depicting the lateral view of the right lower limb. (**A**): The distal reference point is positioned at the center of the lateral aspect of the knee joint line. (**B**): The proximal reference point is positioned one centimeter posterior to the anterior superior iliac spine. (**C**): Tensor fasciae latae and iliotibial tract assessment points are positioned at five equidistant locations, obtained by dividing the total distance into six equal intervals between the reference points.

A measurement was performed by aligning a measuring tape between the center of the lateral aspect of the knee joint line (Fig. 5A) and a reference point positioned one centimeter posterior to the anterior superior iliac spine (Fig. 5B). The tensor fasciae latae and iliotibial tract assessment points were obtained by dividing the total distance between these reference points into six equal intervals, with markings placed at five equidistant locations along this axis (Fig. 5C).

### Assessment protocol for the posterior thigh region

**Fig. 6:**
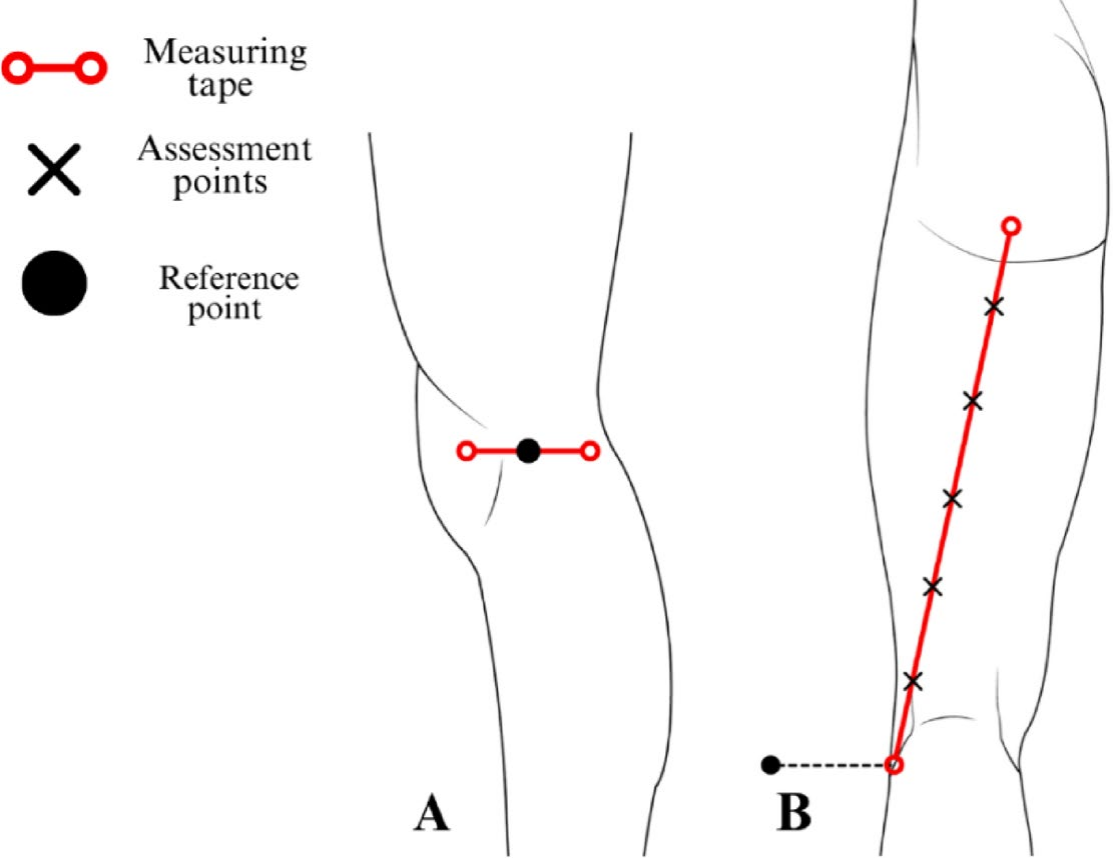
Protocol for mapping assessment points in the posterior thigh region. The figure presents the assessment protocol for the posterior thigh region, depicting the lateral and posterior view of the right lower limb. (**A**): The distal reference point is positioned at the center of the lateral aspect of the knee joint line. (**B**): The proximal reference point is established at the ischial tuberosity, with the biceps femoris assessment points positioned at five equidistant locations, obtained by dividing the total distance into six equal intervals between the reference points.

A measuring tape was aligned between the center of the ischial tuberosity (Fig. 6B) and the lateral aspect of the knee joint line (Fig. 6A). The total distance between these reference points was divided into six equal intervals, with markings placed at five equidistant locations along this axis to establish the biceps femoris assessment points (Fig. 6B).

### Assessment protocol for the posterior leg region

**Fig. 7:**
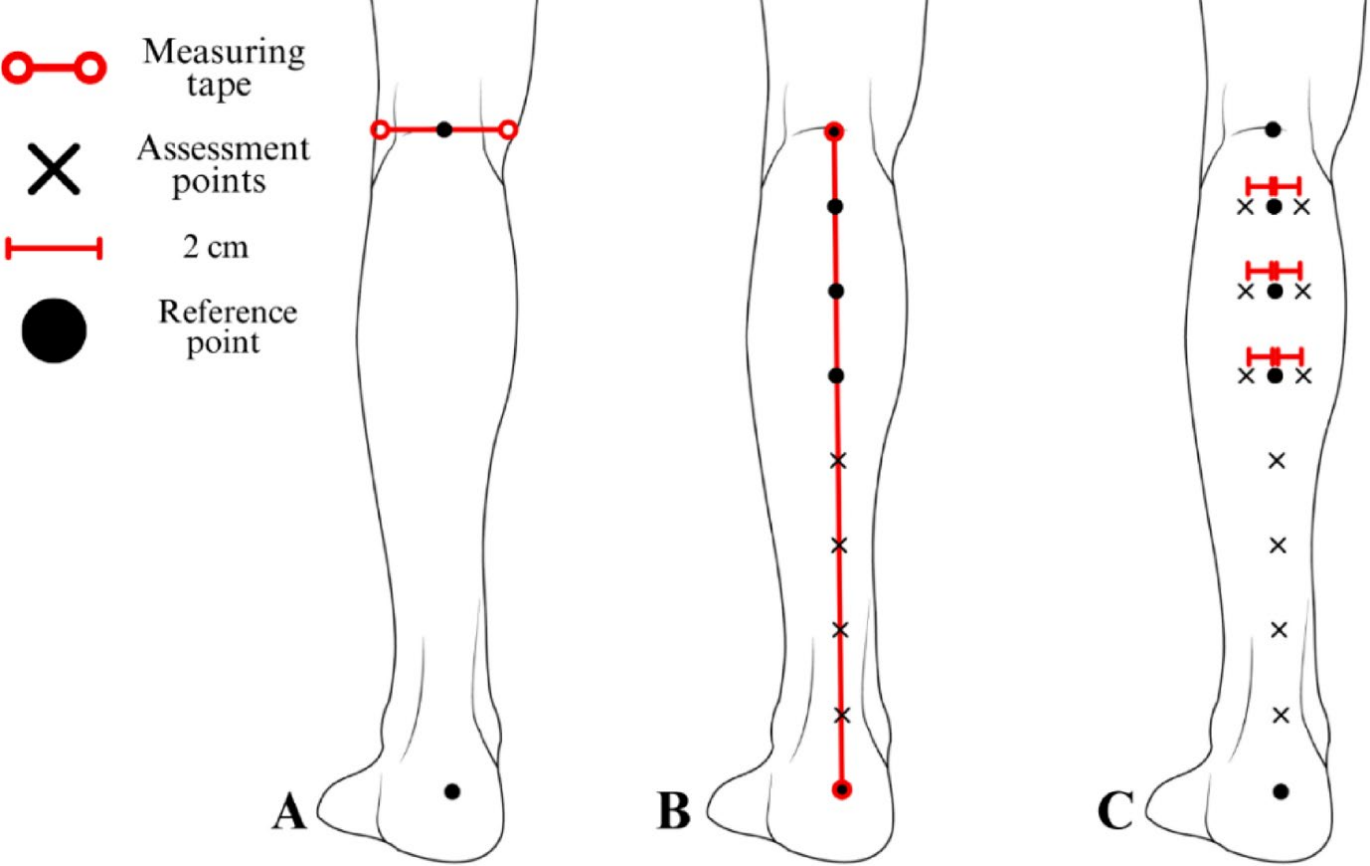
Protocol for mapping assessment points in the posterior leg region. The figure presents the assessment protocol for the posterior leg region, depicting the posterior view of the right lower limb. (**A**): The proximal reference point is established at the center of the popliteal fossa, and the distal reference point is located at the calcaneal tuberosity. (**B**): The gastrocnemius reference points, along with the assessment sites for the calcaneal tendon and the musculotendinous junction of the gastrocnemius and calcaneal tendon, are positioned at seven equidistant locations, obtained by dividing the total distance into eight equal intervals. (**C**): Specific gastrocnemius assessment points are established, with markings positioned two centimeters laterally for the lateral gastrocnemius assessment and two centimeters medially for the medial gastrocnemius assessment.

A measuring tape was aligned between the center of the popliteal fossa and the calcaneal tuberosity (Fig. 7A). The total distance between these reference points was divided into eight equal intervals, with markings placed at seven equidistant locations along this axis (Fig. 7B).

From the three proximais points, additional markings were positioned two centimeters laterally and medially to establish the assessment points for the lateral and medial gastrocnemius, respectively (Fig. 7C). The central point within this segment corresponds to the assessment location for the musculotendinous junction of the gastrocnemius and calcaneal tendon, while the three distal points correspond to the assessment sites for the calcaneal tendon (Fig. 7C).

### Assessment protocol for the plantar region

**Fig. 8:**
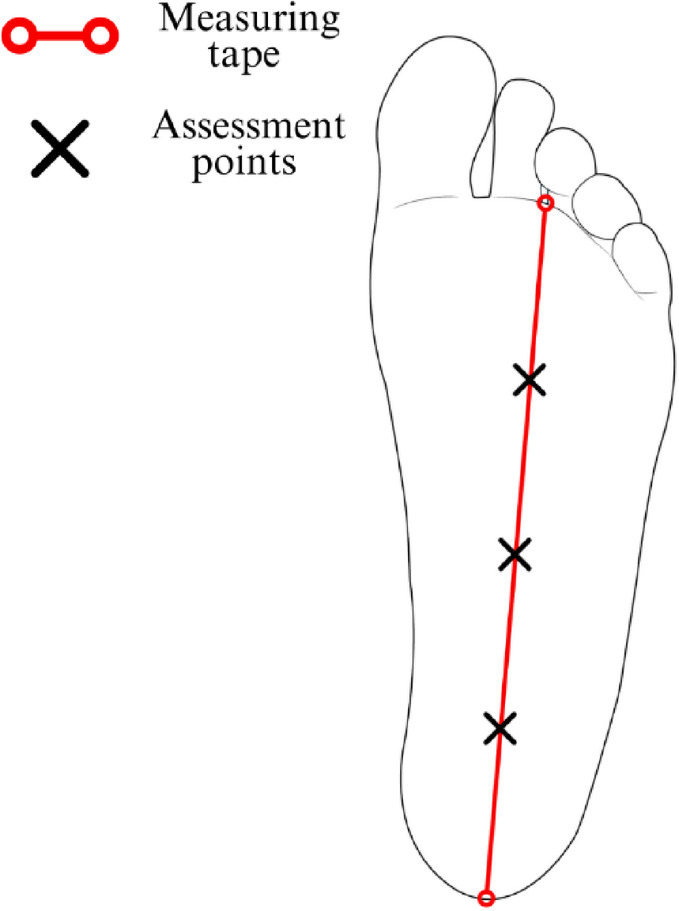
Protocol for mapping assessment points in the plantar fascia. The figure presents the assessment protocol for the plantar fascia region, depicting the inferior view of the right lower limb. Assessment points were established between the calcaneal tuberosity and the base of the second and third toes, positioned at three equidistant locations, obtained by dividing the total distance into four equal intervals between the reference points.

A measuring tape was aligned between the calcaneal tuberosity and the base of the second and third toes. The total distance between these reference points was divided into four equal intervals, with markings placed at three equidistant locations along this axis (Fig. 8).

### Statistical and data processing procedures

Data processing was conducted using IBM^®^ SPSS Statistics 20.0, with a 5% significance level. Bilateral comparisons were performed using the Mann-Whitney U test. The absence of significant differences between limbs permitted data pooling, yielding 26 limbs for analysis.

The values of the five myotonometry variables (Frequency, Stiffness, Decrement, Relaxation, and Creep) provided by the MyotonPRO were described using mean and standard deviation. The relationships between Stiffness (N/m) values ​​observed at consecutive assessment points were analyzed using the Wilcoxon signed-rank test. This non-parametric test was selected due to: (1) the small sample size, which limits confidence in normality assumptions required for parametric tests; (2) the robustness of non-parametric tests to outliers and non-normal distributions common in biomechanical measurements; and (3) the conservative nature appropriate for exploratory methodological studies. Effect sizes were calculated as r = Z/√N, where Z is the standardized test statistic and N is the number of paired observations.

All myotonometry data collected has been made available in an Excel file in the supplementary files section.

### Statistical power considerations

Formal post-hoc power analysis for non-parametric tests presents inherent methodological challenges, as standard power analysis approaches are designed primarily for parametric tests. Direct conversion of effect sizes from Wilcoxon signed-rank tests (r = Z/√N) to parametric effect size measures involve approximations with theoretical limitations. Given these constraints and the bilateral data structure discussed above, we acknowledge that formal power calculations may not fully capture the statistical characteristics of our analyses. However, several factors support the adequacy of our statistical approach: (1) observed effect sizes were consistently large across multiple comparisons (*r* = 0.595–0.874, all *p* < 0.005), substantially exceeding conventional thresholds for large effects (*r* > 0.50)^26^; (2) the repeated measures design with 38 assessment points per limb provides greater statistical sensitivity than simple between-subject comparisons; and (3) our findings converge with previous multi-point studies^7,8,17,19^, suggesting biological signal rather than statistical artifact. Nevertheless, these preliminary findings require validation in larger, adequately powered cohortsstrict independence.

### Data visualization

The mean values obtained were transferred to Microsoft^®^ Excel, where conditional formatting was applied to assign a color intensity to each value. After applying intensity colors based on the proposed scale, Canva^®^ graphic design software was used to create figures integrating the proposed points with the intensity of myotonometry variables.

### Sample characterization

The developed protocol was applied to 13 healthy male volunteers, with a mean age of 32.15 (± 6.50) years, a mean weight of 85.34 (± 16.12) kg, and a mean height of 177.07 (± 5.34) cm. Electrical bioimpedance assessments demonstrated symmetry between limbs, with a mean lean mass of 10.26 (± 1.27) kg in the right lower limb and 10.14 (± 1.24) kg in the left lower limb. Similarly, fat mass measurements indicated 2.49 (± 1.45) kg in the right lower limb and 2.46 (± 1.47) kg in the left lower limb.

## Results

### Bilateral analysis of stiffness

Comparison between the right and left lower limbs demonstrated symmetry across all the assessed points, with no significant differences observed between the limbs at any of the 38 points evaluated.

### Description of myotonometry values

**Fig. 9:**
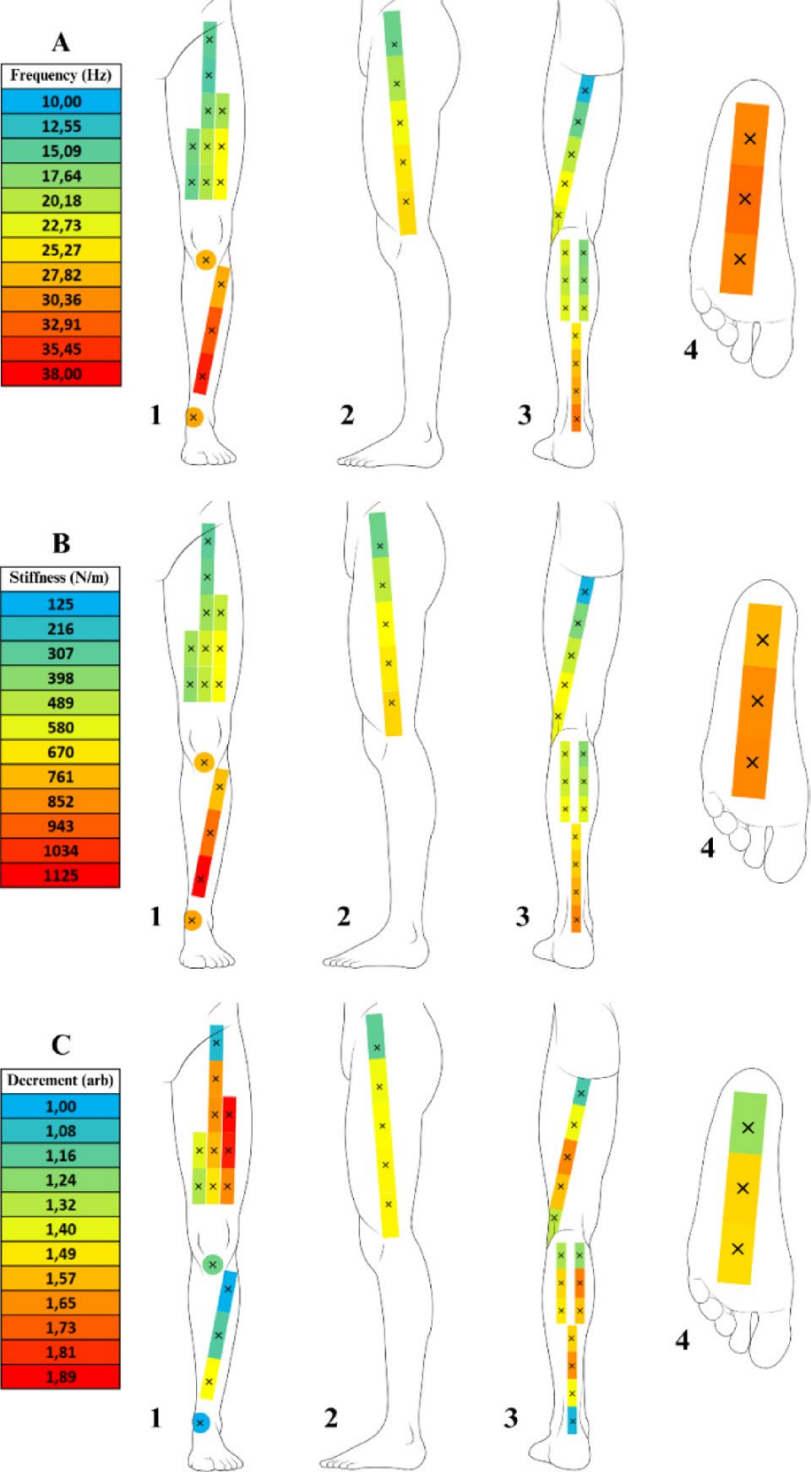
Frequency (Hz), Stiffness (N/m) and Decrement (arb) Values Mapping Across The Assessment Points in the Lower Limbs. The figure presents the distribution of frequency (Hz), stiffness (N/m) and decrement (arb) values through a heat map superimposed on the anatomical structure of the lower limbs, displayed from four distinct perspectives (1 – Anterior view; 2 – Lateral view; 3 – Posterior view; 4 – Inferior view). The chromatic scale represents the magnitude of the variables, progressing from blue (lower values) through green, yellow, and orange, to red (higher values), providing a gradual visualization of variations across assessment points. (**A**) Frequency (Hz); (**B**) Stiffness (N/m); C – Decrement (arb).

**Fig. 10:**
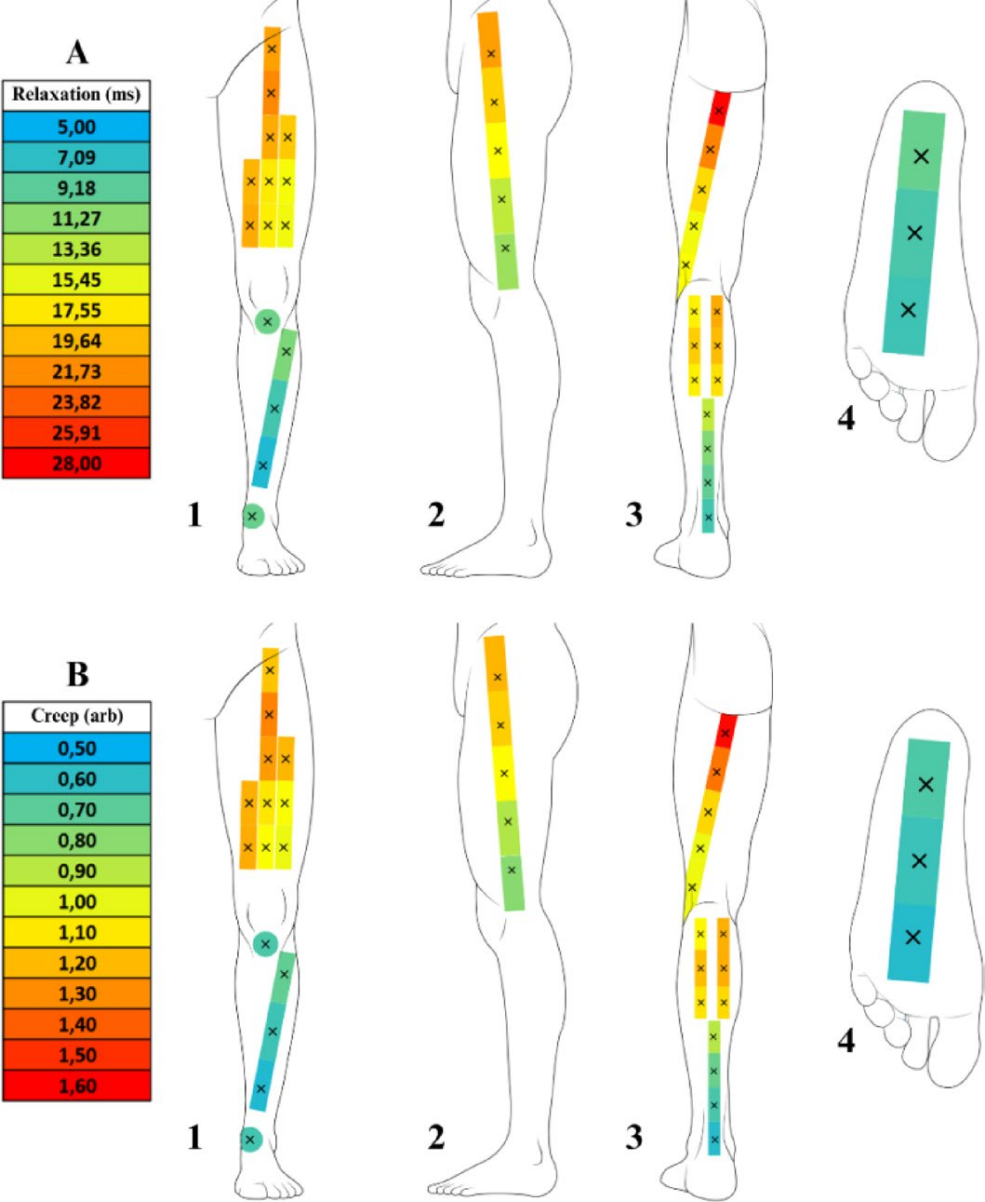
Relaxation (ms) and Creep (arb) Values Mapping Across The Assessment Points in the Lower Limbs. The figure presents the distribution of relaxation (ms) and creep (arb) values through a heat map superimposed on the anatomical structure of the lower limbs, displayed from four distinct perspectives (1 – Anterior view; 2 – Lateral view; 3 – Posterior view; 4 – Inferior view). The chromatic scale represents the magnitude of the variables, progressing from blue (lower values) through green, yellow, and orange, to red (higher values), providing a gradual visualization of variations across assessment points. (**A**) Relaxation (ms); (**B**) Creep (arb).

The mean values and standard deviations for the myotonometry variables — Frequency (Hz), Stiffness (N/m), Decrement (arb), Relaxation (ms), and Creep (arb) — are presented in Table 1. The myotonometry mean values at each assessment point, are presented in Figs. 9 and 10, using a chromatic scale to visually represent the magnitude of values.

**Table 1:** Description of myotonometry values.

Assessment point (n = 26 lower limbs)	Frequency (Hz) x̄ (sd)	Stiffness (N/m) x̄ (sd)	Decrement (arb) x̄ (sd)	Relaxation (ms) x̄ (sd)	Creep (arb) x̄ (sd)
RF 1	12.3 (±1.24)	197 (± 37.8)	1.07 (± 0.16)	21.34 (± 2.85)	1.21 (± 0.15)
RF 2	11.9 (± 1.02)	210 (± 32.1)	1.63 (± 0.22)	22.32 (± 1.83)	1.33 (± 0.10)
RF 3	12.9 (± 0.97)	247 (± 31.2)	1.64 (± 0.31)	20.87 (± 1.64)	1.29 (± 0.09)
RF 4	14.5 (± 1.23)	287 (± 28.1)	1.57 (± 0.32)	18.29 (± 1.42)	1.14 (± 0.08)
RF 5	14.1 (± 1.59)	273 (± 44.0)	1.49 (± 0.32)	18.10 (± 1.68)	1.08 (± 0.07)
VL 3	13.9 (± 1.63)	276 (± 35.2)	1.88 (± 0.33)	19.66 (± 1.86)	1.23 (± 0.10)
VL 4	16.0 (± 2.20)	326 (± 44.5)	1.84 (± 0.34)	17.05 (± 2.17)	1.08 (± 0.13)
VL 5	15.9 (± 2.05)	317 (± 47.8)	1.65 (± 0.29)	16.79 (± 2.31)	1.05 (± 0.13)
VM 4	12.8 (± 0.63)	249 (± 19.7)	1.40 (± 0.21)	20.48 (± 1.28)	1.25 (± 0.08)
VM 5	12.5 (± 0.85)	235 (± 32.0)	1.32 (± 0.14)	20.92 (± 1.84)	1.25 (± 0.11)
IT 1	22.6 (± 4.01)	541 (± 133.1)	1.20 (± 0.14)	10.37 (± 1.64)	0.67 (± 0.07)
TA 1	23.1 (± 3.50)	519 (± 128.9)	1.02 (± 0.18)	11.14 (± 1.78)	0.72 (± 0.09)
TA 2	30.6 (± 5.57)	777 (± 215.4)	1.15 (± 0.30)	8.36 (± 1.39)	0.64 (± 0.06)
TA 3	35.5 (± 5.70)	1118 (± 357.8)	1.45 (± 0.20)	6.59 (± 0.92)	0.57 (± 0.05)
MR 1	23.5 (± 4.55)	606 (± 193.1)	1.02 (± 0.17)	10.09 (± 2.67)	0.66 (± 0.11)
TFLITT 1	12.5 (± 1.41)	209 (± 49.2)	1.18 (± 0.21)	21.38 (± 2.68)	1.24 (± 0.14)
TFLITT 2	14.1 (± 1.41)	272 (± 54.1)	1.43 (± 0.28)	19.29 (± 2.70)	1.19 (± 0.15)
TFLITT 3	15.7 (± 2.01)	319 (± 50.4)	1.45 (± 0.29)	17.42 (± 2.40)	1.09 (± 0.14)
TFLITT 4	17.9 (± 2.58)	383 (± 62.0)	1.46 (± 0.18)	14.39 (± 2.27)	0.91 (± 0.13)
TFLITT 5	19.2 (± 3.94)	453 (± 117.7)	1.46 (± 0.38)	12.66 (± 2.99)	0.81 (± 0.15)
BF 1	10.5 (± 0.81)	137 (± 25.6)	1.14 (± 0.20)	27.80 (± 3.20)	1.58 (± 0.19)
BF 2	12.5 (± 1.30)	221 (± 45.4)	1.43 (± 0.25)	22.51 (± 2.87)	1.37 (± 0.16)
BF 3	14.5 (± 1.49)	279 (± 37.4)	1.66 (± 0.32)	18.92 (± 2.15)	1.17 (± 0.13)
BF 4	16.0 (± 2.28)	321 (± 57.4)	1.56 (± 0.21)	16.88 (± 2.76)	1.05 (± 0.17)
BF 5	15.3 (± 2.96)	309 (± 90.2)	1.34 (± 0.25)	17.28 (± 3.57)	1.05 (± 0.20)
LG 1	15.1 (± 1.46)	291 (± 38.1)	1.31 (± 0.20)	17.97 (± 1.82)	1.10 (± 0.09)
LG 2	14.6 (± 1.43)	285(± 37.2)	1.52 (± 0.36)	19.46 (± 1.87)	1.22 (± 0.11)
LG 3	15.3 (± 1.94)	312 (± 49.4)	1.49 (± 0.25)	18.04 (± 2.52)	1.14 (± 0.14)
MG 1	12.9 (± 0.89)	229 (± 29.9)	1.26 (± 0.22)	20.88 (± 1.72)	1.25 (± 0.10)
MG 2	13.3 (± 1.30)	261 (± 27.3)	1.68 (± 0.41)	20.22 (± 1.36)	1.25 (± 0.08)
MG 3	14.6 (± 1.50)	298 (± 49.5)	1.56 (± 0.29)	18.63 (± 2.44)	1.16 (± 0.15)
GC 1	17.8 (± 2.06)	390 (± 42.4)	1.53 (± 0.29)	14.42 (± 1.60)	0.92 (± 0.09)
CT 1	21.4 (± 2.88)	479 (± 45.5)	1.64 (± 0.28)	11.41 (± 1.13)	0.74 (± 0.06)
CT 2	23.6 (± 3.51)	572 (± 89.0)	1.44 (± 0.22)	9.86 (± 1.26)	0.67 (± 0.05)
CT 3	27.9 (± 2.95)	707 (± 114.7)	1.05 (± 0.14)	8.31 (± 0.97)	0.59 (± 0.04)
PF 1	26.4 (± 2.92)	554 (± 114.7)	1.28 (± 0.13)	10.07 (± 1.45)	0.68 (± 0.07)
PF 2	28.7 (± 2.64)	681 (± 103.9)	1.52 (± 0.15)	8.61 (± 0.93)	0.63 (± 0.05)
PF 3	30.6 (± 2.90)	697 (± 84.9)	1.15 (± 0.12)	8.09 (± 0.81)	0.58 (± 0.04)

### Stiffness (N/m) between consecutive assessment points

#### Anterior thigh region

The rectus femoris showed a statistically significant increase in stiffness from proximal to distal, with differences at RF2 – RF3 (Z = 4.356; *p* = 0.000; *r* = 0.854) and RF3 – RF4 (Z=-4.305; *p* = 0.000; *r* = 0.844). Similarly, the vastus lateralis had a proximal stiffness increase, particularly between VL3 – VL4 (Z=-4.330; *p* = 0.000; *r* = 0.849). The vastus medialis did not show a significant difference between VM4- VM5 (*p* = 0.066). A statistically significant increase in stiffness is observed from the vastus medialis to the rectus femoris, with differences at RF4 – VM4 (Z=-4.432; *p* = 0.000; *r* = 0.869) and RF5 – VM5 (Z=-4.127; *p* = 0.000; *r* = 0.809). A further increase was noted from the rectus femoris to the vastus lateralis, particularly at RF3 – VL3 (Z=-3.822; *p* = 0.000; *r* = 0.750), RF4 – VL4 (Z=-3.978; *p* = 0.000; *r* = 0.780), and RF5 – VL5 (Z=-3.086; *p* = 0.002; *r* = 0.605).

Additionally, stiffness was significantly higher in the vastus medialis, rectus femoris, and vastus lateralis compared to the infrapatellar tendon, with differences at VM5 – IT1 (Z=-4.457; *p* = 0.000; *r* = 0.874), RF5 – IT1 (Z=-4.458; *p* = 0.000; *r* = 0.874), and VL5 – IT1 (Z=-4.458; *p* = 0.000; *r* = 0.874). In the anterior leg region, stiffness significantly increased from proximal to distal between tibialis previous points and from the tibialis anterior to the medial retinaculum, with differences at TA1 – TA2 (Z=-4.432; *p* = 0.000; *r* = 0.869), TA2 – TA3 (Z=-4.203; *p* = 0.000; *r* = 0.824), and TA3 – MR1 (Z=-4.432; *p* = 0.000; *r* = 0.869).

#### Lateral thigh region

The lateral thigh region showed a proximal-to-distal stiffness statistically significant increase across all assessed points: (TFLITT1 – TFLITT2: Z=-4.153; *p* = 0.000; *r* = 0.814; TFLITT2 – TFLITT3: Z=-4.051; *p* = 0.000; *r* = 0.794; TFLITT3 – TFLITT4: Z=-4.254; *p* = 0.000; *r* = 0.834; TFLITT4 – TFLITT5: Z=-3.314; *p* = 0.001; *r* = 0.650).

### Posterior thigh region

The biceps femoris exhibited a statistically significant increase in stiffness from proximal to distal, with differences observed between BF1 – BF2 (Z=-4.457; *p* = 0.000; *r* = 0.874), BF2 – BF3 (Z=-4.458; *p* = 0.000; *r* = 0.874), and BF3 – BF4 (Z=-3.035; *p* = 0.002; *r* = 0.595). In the transition to the leg, a significant increase was noted only between BF5 and MG1 (Z=-3.797; *p* = 0.000; *r* = 0.745).

#### Posterior leg region

The posterior leg region maintained the proximal-to-distal stiffness increase observed previously, showing statistically significant differences between the gastrocnemii and the calcaneal tendon at: LG2 – LG3 (Z=-3.213; *p* = 0.001; *r* = 0.630), LG3 – GC1 (Z=-4.432; *p* = 0.000; *r* = 0.869) MG1 – MG2 (Z=-3.708; *p* = 0.000; *r* = 0.727), MG2 – MG3 (Z=-4.204; *p* = 0.000; *r* = 0.824), MG3 – GC1 (Z=-4.407; *p* = 0.000; *r* = 0.864), GC1 – CT1 (Z=-4.407; *p* = 0.000; *r* = 0.864), CT1 – CT2 (Z=-4.305; *p* = 0.000; *r* = 0.844), and CT2 – CT3 (Z=-4.026; *p* = 0.000; *r* = 0.790). Additionally, a medial-lateral stiffness significant increase was observed between the medial and lateral gastrocnemius, with significant differences at: LG1 – MG1 (Z=-4.407; *p* = 0.000; *r* = 0.864), LG2 – MG2 (Z=-4.051; *p* = 0.000; *r* = 0.794), and LG3 – MG3 (Z=-2.794; *p* = 0.005; *r* = 0.548).

#### Plantar region

In the tendon-fascia transition, a statistically significant reduction in stiffness was observed between the distal point of the calcaneal tendon and the proximal point of the plantar fascia (CT3 – PF1: Z=-3.670; *p* = 0.000; *r* = 0.720). The plantar fascia then exhibited a statistically significant increase in stiffness from proximal to distal at PF1 – PF2 (Z=-4.432; *p* = 0.000; *r* = 0.869).

## Discussion

The present study aimed to establish a standardized and reproducible protocol for evaluating the biomechanical and viscoelastic properties of the lower limbs in healthy adults using myotonometry. This methodology is based on multiple assessment points normalized by individual anatomical length, distinguishing it from previous studies that, although incorporating multiple measurement locations within the same structure^7,8,17–20^, did not implement standardized normalization procedures. This methodological refinement reduces potential biases introduced by manual palpation while establishing comparative parameters with existing literature.

As a methodological development study, the primary contribution of this investigation is the standardized protocol itself, which addresses significant gaps in current myotonometry practice. The preliminary data demonstrate that this protocol can detect statistically significant, location-dependent variations with substantial effect sizes, supporting its potential utility for future research. However, several important methodological considerations contextualize these findings. The effective sample size of 13 independent participants, while adequate for demonstrating the protocol’s discriminative capacity given the large effect sizes observed, elevates risks of Type II errors for subtle effects and limits generalizability. The homogeneous sample (exclusively male participants, narrow age range) further constrains external validity. These limitations do not diminish the protocol’s value as a standardized methodological tool but underscore that establishing robust normative values, clinical thresholds, and broader applicability requires validation in larger, more diverse, adequately powered populations employing appropriate multilevel analytical frameworks.

Statistical analysis revealed significant stiffness variations between assessment points, with high effect sizes^26^. Stiffness (N/m) demonstrated statistically significant increases in both proximal-distal and medial-lateral directions, with progressively higher values observed in the distal and lateral regions. This directional behavior and property transmission reinforce the necessity of multiple-points assessments, further supporting the location-dependent nature of myotonometry variables, as previously reported in the literature^7,8,17,19^. The convergence of our effect sizes and stiffness patterns with independent multi-point studies^7,8,17,19^ reduces the likelihood that the large effects observed here are mere methodological artifacts, supporting a genuine biological signal rather than spurious inflation due to small sample size.

This investigation expands existing knowledge by statistically demonstrating the significance of variations between assessment points, an aspect not previously addressed in earlier studies. The results obtained aligned with findings from Gervasi et al.^17^ and Morgan et al.^19^ regarding stiffness behavior in the calcaneal tendon. Similarly, García-Bernal et al.^7^, who evaluated the calcaneal tendon and gastrocnemii at multiple points, reported stiffness behavior highly consistent with the present study and other prior research on calcaneal tendon properties^17,19^. This convergence of results, despite methodological differences, reinforces not only the general distribution of stiffness but also the presence of specific stiffness variation patterns across anatomical structures.

Methodologically, this study distinguishes itself from previous research^7,8,17–20^ by incorporating comparative statistical analyses across multiple assessment points, identifying significant differences with substantial effect sizes^26^. This approach addresses an important methodological gap in the literature, recognizing that prior studies primarily focused on applications and hypothesis generation^7,8,17–20^. The statistical characterization of location-dependent stiffness variations, combined with prior literature^7,8,17–20^, underscores the potential of stiffness as a clinically relevant biomarker.

Morgan et al.^19^ demonstrated significant differences in the behavior of the five myotonometry variables when comparing individuals with and without calcaneal tendinopathy, while Klich et al.^8^ showed that stiffness appears to adapt to the specific demands of different sports disciplines. Similarly, García-Bernal et al.^7^ reported that health conditions, such as stroke, can alter the behavior of frequency and stiffness throughout the limb.

These potential biomarkers are strongly influenced by individual factors, including sex, age, and specific health conditions^6,17,19,20^. The present study adds the location of assessment as a newly identified determinant influencing myotonometry measurements. However, location-dependent variability in myotonometry values introduces two primary explanatory hypotheses: (1) the potential influences of the perimeter of the assessment site, and (2) the tissue composition within this perimeter.

The first hypothesis is based on mathematical models of stiffness assessment using myotonometry and force platforms, both of which apply the same equation used to calculate the elasticity constant in linear springs^27,28^. However, while linear springs exhibit a single stiffness value throughout the entire structure, non-linear systems display variations in the elasticity constant^27^. In non-linear springs, coil diameter directly influences stiffness, with smaller diameters generating higher stiffness values and larger diameters producing lower stiffness values^27^.

The second hypothesis considers the penetration depth of the myotonometric signal, which is limited to 20 mm^29^, and the influence of deep anatomical structures on recorded stiffness values, as demonstrated by Lim and Choi^20^. Variations in tissue composition and structural architecture throughout the lower limb could modulate stiffness measurements at different locations. These findings highlight the need for future investigations to explore the feasibility of using perimetric or ultrasonographic measurements as complementary normalization variables for myotonometry.

### Limitations of the study

This study has several limitations that constrain interpretation and generalizability. First, the small sample size (*n* = 13 independent participants) represents a fundamental limitation inherent to this methodological development study. While the consistently large effect sizes (*r* > 0.60 for most comparisons) and the repeated measures design provide adequate sensitivity for detecting the systematic variations demonstrated, small samples^24^ intrinsically elevate risks of Type II errors for subtle effects and potential inflation of effect size estimates. The achieved statistical power varied across the observed effect size range: 0.483 at the lower bound (*r* = 0.595), 0.765 at the median (r̃=0.834), and 0.803 at the upper bound (*r* = 0.874). While the lower bound power is suboptimal, this value corresponds to the minimum effect observed, which was atypical among our 37 comparisons. The median effect more accurately represents the protocol’s discriminative capacity, and all p-values were highly significant (*p* < 0.005, majority *p* < 0.001), reducing concern about false positive findings. Future, validation studies require adequately powered designs with sample sizes determined by formal a priori power analysis.

The achieved statistical power at the median observed effect size (r̃ = 0.834) was 0.765, with corresponding values of 0.483 at the lower bound (*r* = 0.595) and 0.803 at the upper bound (*r* = 0.874). These estimates indicate an increased risk of Type II error for smaller effects, a limitation often inherent to exploratory pilot designs. A supplementary sensitivity analysis of statistical power across the observed effect-size range is provided in Supplementary Table S1 and Supplementary Figure S1. Moreover, the all-male, small sample limits generalizability, and future validation studies employing larger, more diverse cohorts are recommended to accurately estimate normative values and strengthen external validity. Accordingly, prospective calculations based on the observed effect sizes indicate that approximately 26–30 independent participants would be required to achieve 80% statistical power (α = 0.05) for detecting large effects (*r* ≈ 0.50), reinforcing the need for larger, adequately powered studies to confirm and extend the preliminary findings reported here.

Second, the homogeneous sample composition (exclusively male participants, narrow age range 25–44 years, limited ethnic diversity) limits generalizability. Sex, age, physical activity level, and body composition are known modulators of tissue mechanical properties^6,17,19,20^, yet our sample precludes evaluation of these factors. Whether the observed location-dependent stiffness patterns generalize across demographic groups, clinical populations, and different body compositions remains unknown and require systematic investigation.

Third, the bilateral data pooling approach, while justified by absence of significant asymmetry and common biomechanical research, introduces statistical dependence that was not formally addressed. Both limbs from each participant share individual characteristics, violating the independence assumption underlying our analytical approach.

Fourth, our findings establish location-dependent variability in stiffness measurements but do not conclusively determine the mechanisms underlying this variability. We proposed two hypotheses: (1) influence of assessment site perimeter, and (2) tissue composition within the measurement depth—but did not empirically test these. The myotonometric signal penetrates approximately 20 mm^29^, potentially capturing contributions from multiple tissue layers with varying mechanical properties. Future studies should integrate perimeter measurements, mapping techniques, and imaging modalities (ultrasound, magnetic resonance imaging, elastography) to characterize tissue composition at depth, correlating biomechanical data with tissue characteristics (proportions of muscle, fat, connective tissue). These studies should test samples with known compositions or anatomical regions with differing compositions to elucidate the anatomical and compositional factors driving site-specific variations, enabling the development of more sophisticated normalization procedures. Fifth, statistical power analysis for non-parametric tests involves methodological complexities not fully resolved in our approach. While the large, consistent effect sizes and convergence with previous literature support the reliability of our findings, formal quantification of statistical power for Wilcoxon tests requires parametric approximations with inherent limitations. This represents an area where methodological standards for non-parametric biomechanical research would benefit from further development.

Although the approach of converting non-parametric effect sizes to parametric equivalents for use in G*Power is methodologically accepted, it involves inherent approximations that may affect the precision of the power estimates. The power calculations, based on parametric assumptions applied to a non-parametric test, should therefore be interpreted as approximate indicators rather than exact values. Additionally, the relatively small sample size (*n* = 13) restricts the stability and generalizability of the power analysis results. These limitations highlight the need for cautious interpretation and suggest that the findings should be complemented with further studies using larger samples and possibly alternative power estimation methods.

Finally, this is a methodological development study with exploratory findings, not a definitive investigation of tissue properties or clinical diagnostic tool. The primary contribution is the standardized protocol demonstrating capacity to detect systematic variations. Establishing normative reference values, clinical thresholds, diagnostic sensitivity and specificity, and therapeutic monitoring utility requires subsequent validation in diverse, well-powered cohorts with appropriate longitudinal and clinical comparison designs.

## Conclusion

This study developed and implemented a new methodological approach for myotonometry assessments, utilizing multiple standardized assessment points in the lower limbs, based on individual anatomical normalization. As a methodological development study with preliminary exploratory findings, the primary contribution is the standardized protocol itself, which addresses significant gaps in current myotonometry practice. This approach enables a more comprehensive and systematic evaluation of tissue properties, addressing limitations in previous protocols that relied on single-point assessments.

The results describe the biomechanical and viscoelastic variables of myotonometry and demonstrate statistically significant variations in stiffness (N/m) throughout the lower limb, revealing specific distribution patterns and tissue behavior. Stiffness values progressively increased in the distal and lateral directions across most evaluated structures. These findings emphasize the importance of standardizing assessment points in myotonometry protocols, given the locational dependence of biomechanical properties. Thus, the proposed protocol represents a significant advancement in non-invasive biomechanical assessment, offering potential for enhanced diagnostic accuracy, improved therapeutic monitoring, and a deeper understanding of tissue adaptation mechanisms across diverse clinical and functional contexts.

While the small sample size (*n* = 13 independent participants) and homogeneous composition limit immediate clinical application and generalizability, the preliminary findings demonstrate the protocol’s capacity to detect systematic, location-dependent variations in tissue stiffness with large effect sizes. These hypothesis-generating results provide foundation for future adequately powered investigations across diverse populations and clinical conditions. Validation studies employing multilevel analytical frameworks, larger samples, and integration with imaging modalities will be essential for establishing normative reference values, clinical thresholds, and diagnostic utility. The standardized protocol developed herein provides the methodological foundation for such investigations, representing a significant advancement in non-invasive biomechanical assessment methodology.

## Supplementary Information

Below is the link to the electronic supplementary material.

Supplementary Material 1

Supplementary Material 2

## Data Availability

All myotonometry data collected has been made available in an Excel file in the supplementary files section.

## Abbreviations

N/m: Newtons per meter
Hz: Hertz
ms: Millisecond
arb: Arbitrary unit of measurement
SPSS: Statistical package for the social sciences
CAAE: Ethical approval registration number
IBM®: International business machines corporation (referring to IBM SPSS Statistics 20.0 software)
MR1: Medial retinaculum 1
RF: Rectus femoris
VL: Vastus lateralis
VM: Vastus medialis
IT: Infrapatellar tendon
TA: Tibialis anterior
MR: Medial retinaculum
TFLITT: Tensor fasciae latae and iliotibial tract
BF: Biceps femoris
LG: Lateral gastrocnemius
MG: Medial gastrocnemius
GC: Musculotendinous junction between gastrocnemius and calcaneal tendon
CT: Calcaneal tendon
PF: Plantar fascia
