# Supplementary material for: Development of a multi-point mapping protocol for myotonometric assessment: a methodological pilot study
Source: Sci Rep. 2026 Jan 7;16:4852. doi: 10.1038/s41598-025-34869-5 (PMC12873364; doi:10.1038/s41598-025-34869-5)
Supplement: Supplementary file 1 — Supplementary Material 1 [file 41598_2025_34869_MOESM1_ESM.docx]

**Supplementary Table S1. Post-hoc power analysis for Wilcoxon signed-rank test (two-tailed, α = 0.05, n = 13).**

| **Scenario** | **Effect size (r)** | **Converted Cohen’s d (approx.)*** | **Achieved Power (G*Power; Wilcoxon)** | **Notes** |
| --- | --- | --- | --- | --- |
| Lower bound (observed) | 0.595 | 1.481 | 0.483 | Most conservative scenario among our comparisons |
| Median (r̃) | 0.834 | 3.023 | 0.765 | Robust summary of typical effect magnitude |
| Upper bound (observed) | 0.874 | 3.597 | 0.803 | Largest observed effect size |

** Effect sizes (r) were converted to approximate Cohen's d using the formula d = 2r/√(1−r²), following established conventions for inputting non-parametric effect sizes into G*Power (Cohen, 1988; Rosenthal, 1991). While this conversion involves parametric approximations, it provides conservative estimates suitable for power analysis.*

**Supplementary Figure S1.** G*Power 3.1.9.7 input/output screenshots for the Wilcoxon signed-rank test (matched pairs, two-tailed, α = 0.05; n = 13) at three representative effect sizes: (A) r = 0.595 (lower bound), (B) r̃ = 0.834 (median), and (C) r = 0.874 (upper bound). Each panel shows the exact parameters entered and the corresponding achieved power estimate. Screenshots support the sensitivity analysis reported in Supplementary Table S1.

| (A) Lower bound  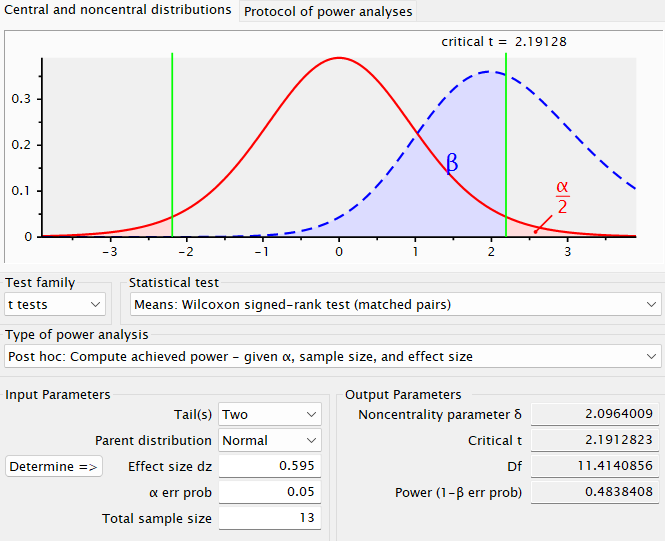 | (B) Median  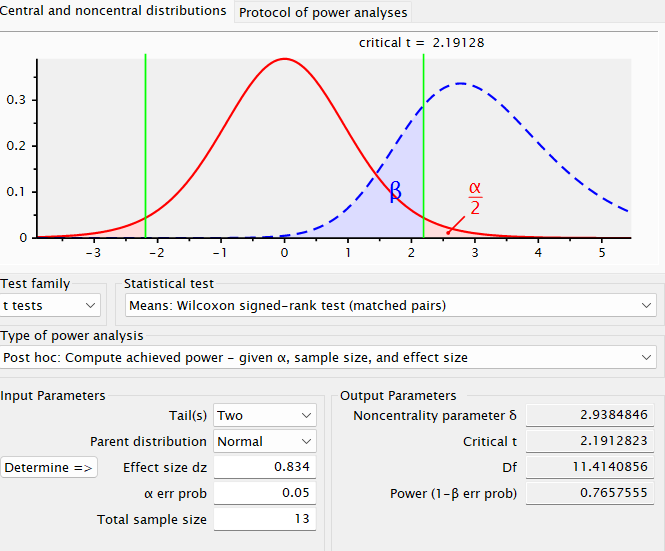 | (C) Upper Bound  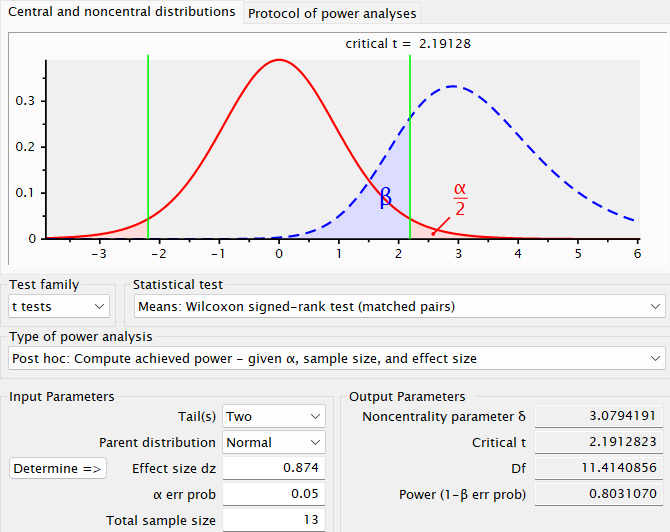 |
| --- | --- | --- |
